# FACTORS ASSOCIATED WITH INFANT MORTALITY IN A BRAZILIAN CITY WITH HIGH HUMAN DEVELOPMENT INDEX

**DOI:** 10.1590/1984-0462/;2017;35;4;00006

**Published:** 2017-09-21

**Authors:** Maria Volpato Kropiwiec, Selma Cristina Franco, Augusto Randüz do Amaral

**Affiliations:** a Universidade Univille, Joinville, SC, Brasil.

**Keywords:** Infant mortality, Risk factors, Economic development, Cohort studies, Mortalidade infantil, Fatores de risco, Desenvolvimento econômico, Estudo de coorte

## Abstract

**Objective:**

To identify factors associated with infant mortality in a city with good socioeconomic development.

**Methods:**

A retrospective cohort study with 7,887 live births in the year of 2012 recorded in the Live Births Information System (SINASC) and associated by linkage with the Mortality Information System (SIM) to identify the deaths in the first year of life. The risk factors were ranked in three levels of determination: distal, intermediate and proximal. The logistic binomial regression models and the multivariate model quantified the impact of the individual variables tested and adjusted the effect of confounding variables. The magnitude of the effect of the explanatory variables was estimated by calculating the crude and adjusted Odds Ratio (OR) and their respective 95% confidence intervals (95%CI), being significant *p*<0.05.

**Results:**

There were 61 deaths in the cohort and the infant mortality rate was 7.7 per thousand live births. Teenage mother (adjOR 3.75; 95%CI 1.40-10.02), gestational age <32 weeks (adjOR 12.08; 95%CI 2.30-63.38), weight at birth <1500g (adjOR 8.20; 95%CI 1.52-44.23), Apgar score at 1 and 5 minutes of life <7 (adjOR 4.82; 95%CI 2.01-11.55 and adjOR 6.26; 95%CI 1,93-20,30, respectively) and the presence of congenital malformation (adjOR 21.49; 95%CI 7.72-59.82) were risk factors for infant mortality.

**Conclusions:**

The lower relevance of socioeconomic and health care variables and the greater importance of biological factors in determining infant mortality may reflect the protective effect of high economic and social development of the locality.

## RESUMO

**Objetivo:**

Identificar os fatores associados à mortalidade infantil em município com bom desenvolvimento socioeconômico.

**Métodos:**

Estudo de coorte retrospectivo com 7.887 nascidos vivos do ano de 2012 registrados no Sistema de Informação sobre Nascidos Vivos (SINASC) e associados por meio de *linkage* com o Sistema de Informações sobre Mortalidade (SIM) para identificação dos óbitos ocorridos no primeiro ano de vida. Os fatores de risco foram hierarquizados em três níveis de determinação: distal, intermediário e proximal. Os modelos de regressão logística binomial e o modelo multivariado quantificaram o impacto individual das variáveis testadas e ajustaram o efeito das variáveis de confundimento. A magnitude do efeito das variáveis explicativas foi estimada pelo cálculo do *Odds Ratio* (OR) bruta e ajustada e de seus respectivos intervalos de confiança de 95% (IC95%), sendo significante *p*<0,05.

**Resultados:**

Identificou-se 61 óbitos na coorte e o coeficiente de mortalidade infantil foi de 7,7 por mil nascidos vivos. Mãe adolescente (ORaj 3,75; IC95% 1,40-10,02), duração da gestação <32 semanas (ORaj 12,08; IC95% 2,30-63,38), peso ao nascer <1.500 g (ORaj 8,20; IC95% 1,52-44,23), Apgar no 1º e no 5º minuto de vida <7 (ORaj 4,82; IC95% 2,01-11,55 e ORaj 6,26; IC95% 1,93-20,30, respectivamente) e presença de malformação congênita (ORaj 21,49; IC95% 7,72-59,82) constituíram fatores de risco para os óbitos.

**Conclusões:**

A menor relevância dos fatores socioeconômicos e assistenciais e a maior importância dos fatores biológicos na determinação dos óbitos infantis podem refletir o efeito protetor do elevado desenvolvimento econômico e social dessa localidade.

## INTRODUCTION

The infant mortality rate is used internationally as the indicator that best shows the stage of economic and social development of a country or region, especially because it has a direct relationship with socioeconomic variables, and, therefore, is sensitive to their variations.^1^ ^,^ ^2^

According to the World Health Organization (WHO), the infant mortality rate allows analyzing the availability, the use and the efficacy of health care, especially care addressed to prenatal, birth, newborn and infant in the first year of life. It is frequently used to define public policies addressed to mother-child health.^2^ ^,^ ^3^

Despite the advancements seen worldwide due to commitments taken over by international institutions, the organized civil society and public policies from several countries, it is still possible to see great disparity in the infant mortality rate between developed and developing countries.^4^ Nowadays, the lowest infant mortality rates are from countries with high Human Development Index (HDI) rates - with three deaths per one thousand live births -, whereas infant mortality rates in countries with low HDI are still high.^5^

Brazil has seen an important reduction in infant mortality throughout the past decades,^3^ due to the decreasing fertility, expanded basic sanitation, reorganization of the health care model (Family Health Strategy - FHS), improvements in child health care, increasing coverage of vaccination campaigns and prevalence of maternal breastfeeding, which influenced the reduction of infectious diseases in the first years of life.^6^ ^,^ ^7^Besides, there was a combination of economic growth and improved schooling and income distribution.^8^

Still, regional inequalities and inequities related with social groups that are considered vulnerable constitute major challenges in our country.^1^ In 2013, of the five Brazilian regions, only the North and the Northeast presented infant mortality rates higher than the goal of 15.7 deaths/one thousand live births, proposed by the Millennium Development Goals (MDG) for 2015.^9^ However, even in the regions where infant mortality reached rates lower than two digits, there were high proportions of deaths considered to be preventable, whose reduction constitutes an opportunity to reach rates close to those in developed countries.^6^ Getting to know the determinants of infant mortality in Brazilian cities whose socioeconomic contexts show good social and health indicators may subsidize interventions in the scope of public health, aiming at its reduction, once such determinants may represent risks of different magnitudes in relation to those observed in less developed regions. 

With an estimated population of 526,338 inhabitants,^10^ the city of Joinville constitutes the third industrial pole in the South region and has one of the highest Gross Domestic Product (GDP) levels in the country. This level of economic development translates into good social and health indicators. In 2010, the city presented with one of the highest Municipal Human Development Index (MHDI) (0.809) among Brazilian cities, in the 21^st^ national position.^8^ ^,^ ^11^ The infant mortality rate has been below two digits since 2001, with an average of 8.7 deaths/one thousand live births in the period.^10^ Considering the exposed, the objective of this study is to measure the infant mortality coefficient and to identify its associated factors in a Brazilian city with high MHDI.

## METHOD

Cohort, retrospective and population-base study including the total number of live births in 2012, residents of the city of Joinville, Santa Catarina, and infant deaths related with this cohort.

The data in the study came from the National Mortality System (SIM) and the Live Birth Information System (SINASC). To identify the deaths related with the cohort, the Federal Infant and Fetal Death Investigation Module was used, which automatically pairs the infant death declarations (DD) and their respective birth declarations (BD), based on the BD number. All DDs and BDs related were manually checked, considering variables that were common to both forms. Besides these, the National Register of Health Establishments (CNES) was used to classify the type and complexity of the establishment, and the Annual Management Report was adopted to categorize the two basic care models (FHS and non-FHS). The data were provided by the Epidemiological Surveillance Management, in charge of birth and death data bases from the Municipal Secretariat of Health in Joinville, Santa Catarina.

After pairing, the survival of the live birth cohort was defined, identifying those who evolved to infant death in the first year of life.

The analysis of infant death (younger than 1 year of age) was conducted according to distal (social), intermediate (care) and proximal (biological) factors, according to the model proposed by Mosley and Chen^12^ in 1984. The distal factors included ethnicity, schooling, occupation and maternal marital status. The intermediate factors included birth in another city, basic care model, place of birth, type of establishment (considering as private the hospitals which assisted private and insurance patients; state-run hospitals, under state management; and social organizations, which exclusively worked with the Unified Health System), hospital complexity (presence of neonatal intensive care unit (ICU), number of dead children, maternal parity, month when the prenatal began, Apgar index at 1 and 5 minutes, and presence of malformations.

All information was treated statistically using the software Statistical Package for the Social Sciences (SPSS), version 21.0 (SPSS Inc., Chicago, Illinois, USA). The qualitative variables were presented in absolute and relative frequencies. Binomial logistic regression models were built to examine the influence of distal, intermediate and proximal factors about infant mortality. The multivariate model was chosen to quantify the individual impact of the tested variables and to adjust the effect of the confounding variables. The magnitude of the effect of explanatory variables was estimated by the calculation of the odds ratio (OR), both crude and adjusted, with its respective 95% confidence intervals (95%CI). OR was used as an approximation of relative risk, given the small number of events and the option to use non-conditional logistic regression to adjust the potential confounding effects. *p*<0,05 was considered significant.

The study was approved by the Research Ethics Council according to report n. 875.237.

## RESULTS

According to SINASC, the total number of live births of mothers living in the city, in 2012, was 7,887 children. In the Federal Infant and Fetal Investigation Module, 123 infant deaths were registered as residents of the city of Joinville from January 1^st^, 2012, to December 31^st^, 2013. Of these, 61 belonged to the cohort of live births from 2012, and, for 100% of them, a linkage was conducted between DD and BD. The infant mortality rate for the cohort was 7.7 deaths per one thousand live births, with prevalence of the neonatal component: 4.4 deaths per one thousand live births. Among the deaths, 35 (57.4%) occurred in the neonatal period, and 26 (42.6%) in the post-neonatal period. Among the neonatal deaths, 20 (32.8%) took place in the early neonatal period (0 to 6 days of life), and 15 (24.6%) in the late neonatal period (7 to 27 days of life).

None of the distal (socioeconomic) characteristics analyzed was associated with the outcome, as presented in Table 1. In the analysis of intermediate factors (care), the variables “birth in a city different than that of residence”, “place of birth”, and “number of prenatal appointments <7”, which had statistical association in the univariate analysis, no longer had it after the multivariate analysis adjustment (Table 2). As to proximal (biological) factors, there was a significant association between most of the studied variables and infant death. After the multivariate analysis, which adjusted the effect of each variable for the effect of the others, there was a reduction of the independent effect of all variables, except for maternal age ≤19 years. In this group, mothers aged ≤19 years had 3.75 more chances (95%CI 1.40-10.02) of death among their newborns. The variable that mostly increased the chances of death was the presence of congenital malformation (OR 21.49), followed by gestational age <32 weeks (OR 12.08), weight at birth <1500 g (OR 8.20), Apgar <7 at 5 minutes (OR 6.26) and at 1 minute (OR 4.82) (Table 3).

**Table 1:**
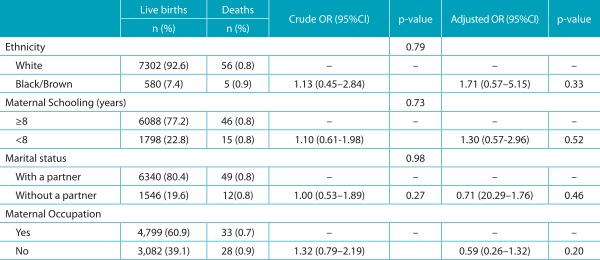
Distribution of absolute and relative frequency, crude and adjusted Odds Ratio in Joinville, Santa Catarina, 2012, according to the distal characteristics.

*The n variation is due to the lack of records in SINASC/SIM; OR: *odds ratio*; CI: confidence interval. Source: SINASC/SIM/CPMI.

**Table 2:**
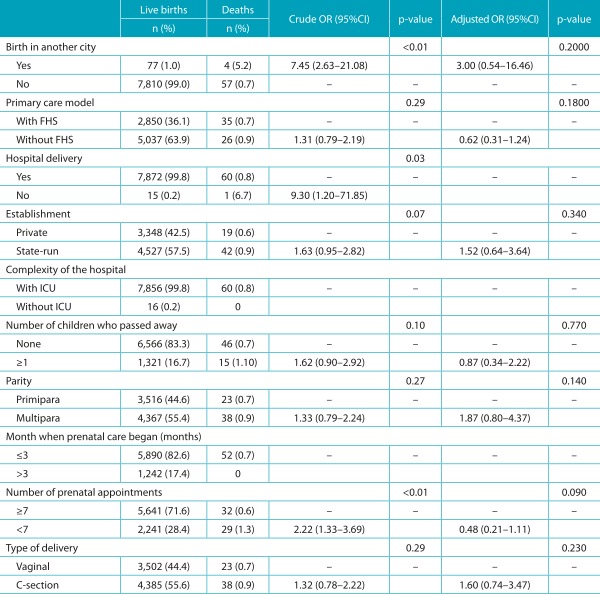
Distribution of absolute and relative frequency, crude and adjusted Odds Ratio in Joinville, Santa Catarina, 2012, according to the intermediate characteristics.

*The n variation is due to the lack of records in SINASC/SIM; OR: *odds ratio*; CI: confidence interval; FHS: Family Health Strategy; ICU: Intensive Care Unit. Source: SINASC/SIM/CPMI.

**Table 3:**
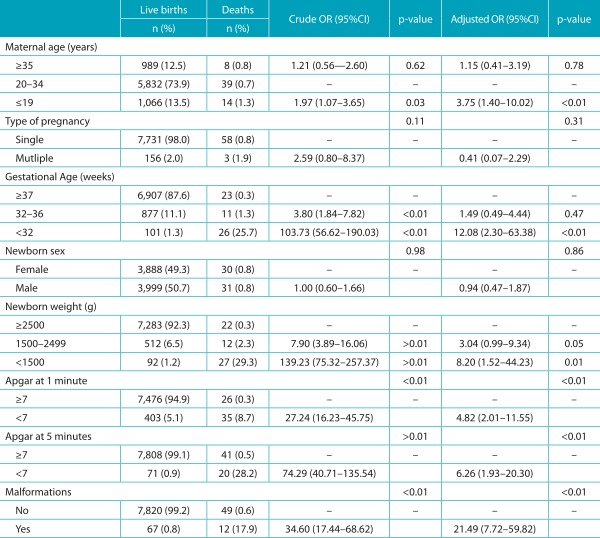
Distribution of absolute and relative frequency, crude and adjusted Odds Ratio in Joinville, Santa Catarina, 2012, according to the proximal characteristics.

*The n variation is due to the lack of records in SINASC/SIM; OR: *odds ratio*; CI: confidence interval. Source: SINASC/SIM/CPMI.

## DISCUSSION

This study verified an infant mortality coefficient considered low (7.7 per one thousand live births), in comparison to the Brazilian reality. The prevalence of the neonatal component, especially the early one, shows a profile that is similar to that of developed regions.^5^ In the city where the study was carried out, several indicators of inequality, income and work are placed in much better levels than the national average.^13^ Some examples are the case of the Gini Index (Joinville 0,49 versus 0.60), mean household income per capita (Joinville R$ 1,114.36 versus Brazil R$ 767.02), PIB per capita (Joinville R$ 40,183.13 versus Brazil R$ 26,445.72), and unemployment rate among people aged more than 16 years (Joinville 4.57% versus Brazil 7.42%), among others.^13^ Even though the infant mortality coefficient is lower than the national average, there is still great potential for reduction, if we consider the high proportion of preventable infant deaths (82%)^10^ - and, with this reduction, the index could be closer to that of more developed countries.

The limitations of this study refer to the use of secondary data sources (DD and BD), which may present some imprecisions, and the restriction of the list of variables of interest for epidemiological studies. Besides, the fact that the sample was small may have influenced data precision, as verified in the amplitude of the OR confidence intervals. The high linkage percentage shows good quality in the filling out of variables in the systems of information used, and the 100% coverage of SINASC and SIM in the city provides the study with a population base.

 In this study, factors traditionally considered as risks for infant mortality, both of socioeconomic (distal) nature and those related with care provided to the mother and to the newborn (intermediate)^14^ ^,^ ^15^ did not increase the chance of death. It is possible that these differences are owed to the characteristics of the locations where the studies were conducted. In Joinville, Santa Catarina, the interaction between well-structured public policies, work opportunities and high economic and social development provide the proper conditions to minimize the effect of social inequalities.^10^ The life condition in this city, when compared to the Brazilian reality, shows a possible protective influence of the socioeconomic context over infant mortality, reducing the effect of maternal and care socioeconomic characteristics.

In this sense, maternal schooling lower than eight years, maternal marital status and maternal work outside the household, unlike other studies, did not increase the chances of infant death.^14^ ^,^ ^16^ ^,^ ^17^ It is worth to mention that our sample was composed mostly of white mothers, with high schooling, married or in a stable union.

It is known that social determinants may produce or strengthen inequities in the access qualified health services.^14^ ^,^ ^18^ In our study, some indicators, such as high levels of hospital delivery (99.8%) and pregnant women who attended the proper number of prenatal care appointments (71.6%), besides the low percentage of births in other cities (1.0%), are indicators of the universal access to health services and the good quality of care. It is important to mention that this study did not check other aspects of care quality, such as the adaptation of work processes, medical conduct, functioning of care flows between the services and the conduction of prenatal examinations, which can contribute with the maintenance of death rates classified as preventable.

The insufficient number of prenatal appointments among the deaths (47.5%) can be caused by the high percentage of extreme premature babies with less than seven appointments who passed away (76.9%), and did not have the opportunity to complete the minimum recommended number of seven appointments. ^19^ Even though we did not show higher chances of infant death among pregnant women who began prenatal care late, or who attended an insufficient number of appointments, these variables are essential for positive neonatal outcomes.^19^ These results, which are apparently controversial, show the complexity of the interaction between socioeconomic aspects and the quality of care provided to the pregnant woman and to the newborn in services with different characteristics, as is the case of public and private services, analyzed together.^20^

This study showed that motherhood during teenage years, prematurity, very low weight at birth, low vitality of the neonate and presence of congenital malformations increased the chances of infant deaths, as is consensually shown in studies from different locations.^14^ ^,^ ^15^ ^,^ ^17^ ^,^ ^18^ ^,^ ^21^ ^,^ ^22^ ^,^ ^23^ ^,^ ^24^ ^,^ ^25^

In the city analyzed, the presence of congenital malformations was the variable that presented the most significant association with infant death. In developed regions, a similar profile was observed due to the control of mortality for other causes.^7^ ^,^ ^26^ In Joinville, even though the magnitude of this problem has not been high among live births (0.8%), it represented the second cause of infant death.^10^ Its cumulative presence requires multidisciplinary and long curative actions, and may lead to financial overload for the health system.^26^ Therefore, actions to prevent malformations, whenever possible, are highly recommended in the public health scope, especially addressing the improvement of prenatal care, the increasing offer of preconception examinations and the genetic counselling for parents.^26^

The low weight at birth (<2500 g) and prematurity (<37 weeks) are recognized as relevant factors for infant death, especially early neonatal death.^4^ ^,^ ^17^ ^,^ ^19^ ^,^ ^23^ ^,^ ^24^ In the studied cohort, pregnancy shorter than 32 weeks (42.6%) and weight below 1500 g (44.2%) showed an association with infant death, suggesting there is an interaction between these two variables, whereas the evolution of pregnancy is followed by the progressive increase in the weight of the newborn. Despite the association between these two factors, in this study prematurity lower than 32 weeks caused more chances of infant death (12 times) than very low weight at birth (8 times).^19^ These aspects are directly related with maternal conditions and prenatal care, which, in turn, work on several determinants and conditions of infant mortality, potentially reducible due to adequate prenatal care.^17^ ^,^ ^19^ ^,^ ^24^

The low vitality of newborns, measured by the Apgar Index, is a factor pointed out by several studies^15^ ^,^ ^17^ ^,^ ^21^ ^,^ ^22^ ^,^ ^24^, corroborated in this study (OR 4.82 and 6.26 at 1 and 5 minutes, respectively) as a predictor of neonatal mortality. This finding reinforces the importance of an adequate surveillance of deliveries and qualified care addressed to the newborn as a way to reduce infant morbimortality resulting from hypoxia.^22^ ^,^ ^24^ Apgar lower than seven is aggravated by the presence of prematurity, low weight and malformations.^15^ ^,^ ^22^ ^,^ ^24^

In Joinville, there were more chances of infant death in children of adolescent mothers (OR 3.75). The relation between maternal age and infant death is not totally clear and needs confirmation.^19^ ^,^ ^25^ Studies show controversial results as to the risk among adolescent mothers,^4^ ^,^ ^14^ ^,^ ^15^ ^,^ ^17^ ^,^ ^18^ ^,^ ^21^ ^,^ ^22^ ^,^ ^23^ ^,^ ^24^ ^,^ ^25^ ^,^ ^27^ indicating that biological factors related with the mother’s young age are mixed with life conditions, especially in cases of low social insertion, thus influencing the reproductive behavior and the morbimortality of children.^28^ The highest prevalence of low schooling, the fact of being single, not having a paid job, starting prenatal care too late, having attended fewer prenatal appointments and urinary infections among adolescent mothers show the complexity of interactions between social conditions and the use of health services with effects on the prevalence of negative neonatal outcomes.^28^

It is important to mention that the biological characteristics of newborns are also affected by the quality of care provided to pregnant women in the pre-delivery period and during labor, and to the newborns as well.^3^ ^,^ ^4^ ^,^ ^18^ It is known that the reduction of neonatal mortality is very sensitive to interventions that depend on health technologies.^3^ ^,^ ^16^ Therefore, the access to hospitals with neonatal ICU, which offer good conditions of physical structure and equipment, with examinations to support the diagnosis and professional teams that are composed of enough people, with trained members, form a set of necessary requirements for the handling of the risk patients, especially extreme premature babies or those with very low weight at birth.^29^ In this study, all hospitals had ICUs, so it was not possible to discriminate this effect.

In the case of state-run hospitals, the high costs to implement and maintain these highly-qualified services properly constitute a critical point to be faced by the administrators, facing the several other demands in the health field. Unlike neonatal specialized care, the offer of qualified health care in other levels of the health system can also cause a positive impact on the reduction of preventable mortality, without requiring expressive investments in technology.^20^ Cities with good level of development and well-structured public policies, in general, have a basic network with good structure, broad population coverage and adequate organization. Therefore, the focus should be on improving the work processes of professionals to quality mother-child care, preventing the birth of vulnerable children with high risk.^29^

This study did not aim at investigating differentials in the profile of patients and in the type of care provided, especially regarding neonatal technologies, for patients using the public health system (SUS) or the private institutions. Therefore, other studies are required to help elucidate the causes of the risk differentials found here. Evaluation studies focusing especially on the work processes may contribute to identify possible factors associated with preventable child death, such as technical quality of the care provided, opportunity to access specialized procedures, among others. Finally, it is important to mention that the occurrence of preventable infant deaths is unacceptable, and requires actions from the society and health policy administrators in all administrative spheres, with the objective of reaching better health levels, getting closer to developed countries. The knowledge of factors associated with infant deaths may subsidize the improvement of public policies, thus contributing in this sense.
